# Supplementary figures and images for: Interleukin-12 elicits a non-canonical response in B16 melanoma cells to enhance survival
Source: Cell Commun Signal. 2020 May 25;18:78. doi: 10.1186/s12964-020-00547-4 (PMC7249691; doi:10.1186/s12964-020-00547-4)

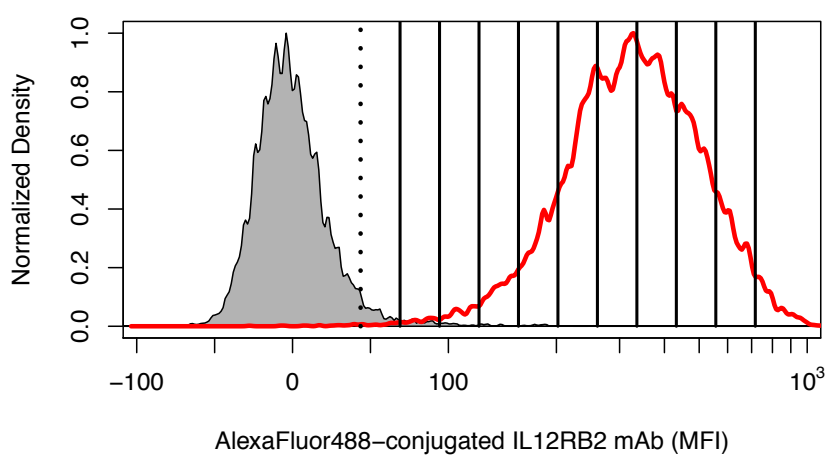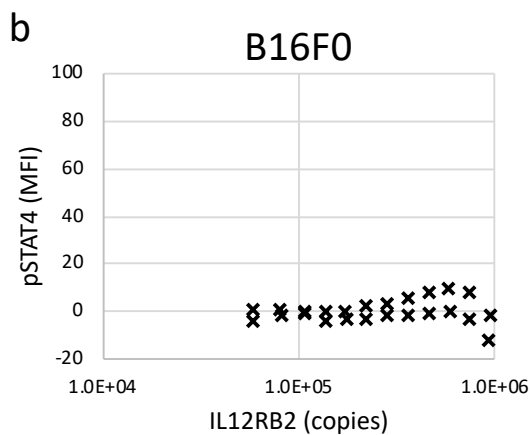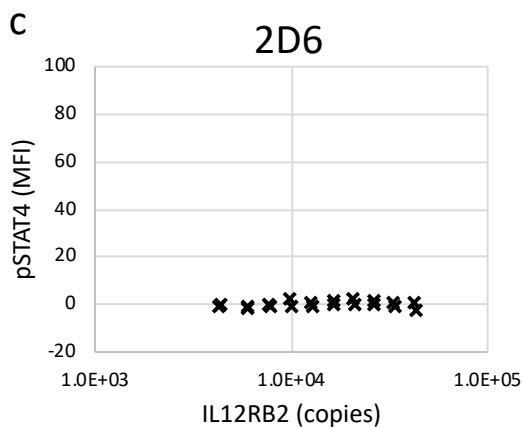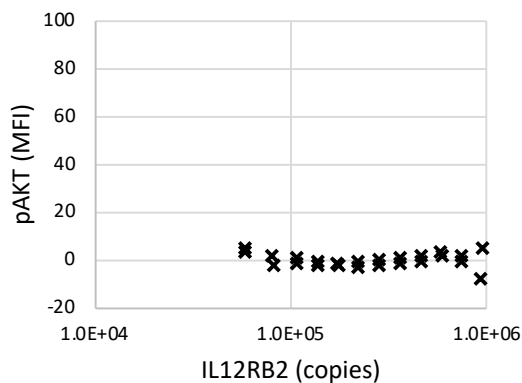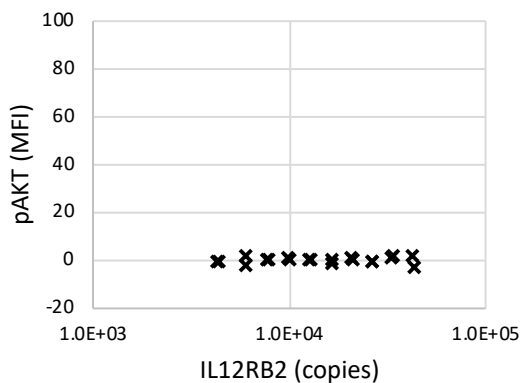

Supplement: Supplementary file 2 — Additional file 1 Supplemental Figure S1: cell subsets were established that vary in expression of iL12RB2 and with minimal fluorescent spillover into pAkt and pSTAT4 measures. (a) Flow cytometric events were subdivided into groups based on IL12RB2 expression, where solid vertical lines denote limits of IL12RB2 expression associated with each subgroup. Red curve denotes distribution in IL12RB2 expression by B16F0 while gray shaded indicates unstained controls. Single-stained controls for IL12RB2 in B16F0 (b) and 2D6 (c) cells were used to establish that fluorescence associated with measuring Akt and STAT4 phosphorylation was not due to fluorescent spillover. Results for two independent experiments are shown for each subgroup based on IL12RB2 expression. [file 12964_2020_547_MOESM1_ESM.pdf]

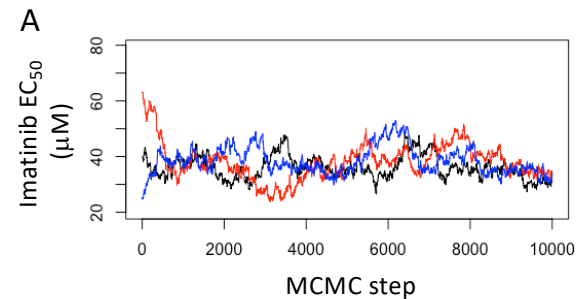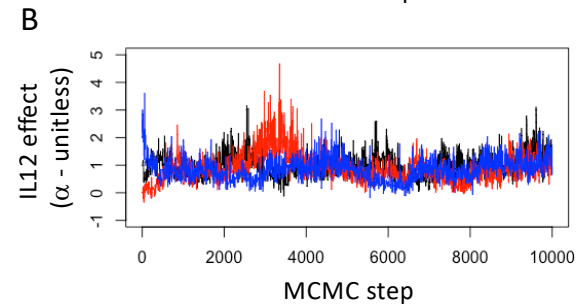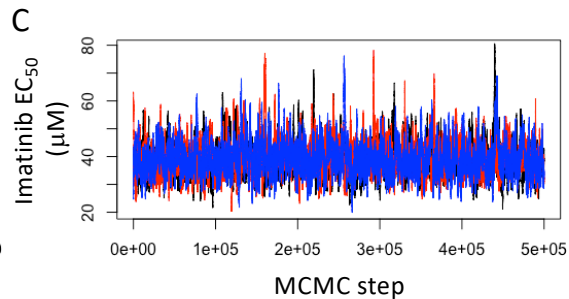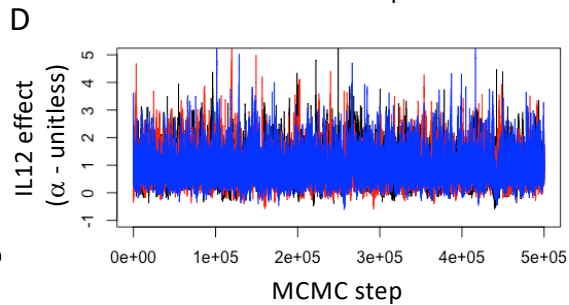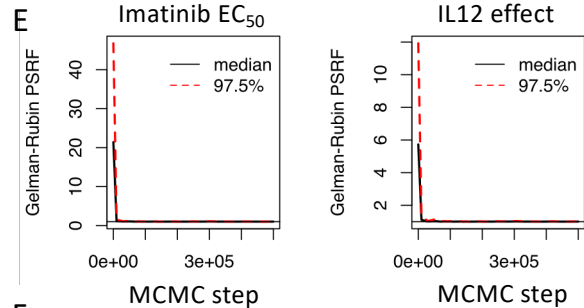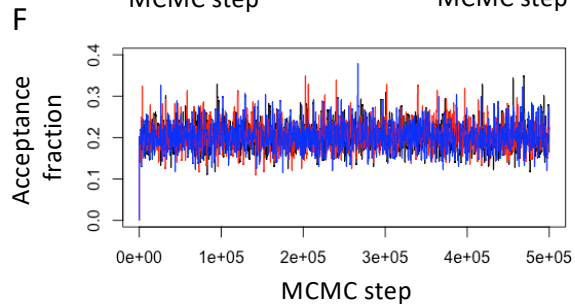

Supplement: Supplementary file 3 — Additional file 2 Supplemental Figure S2: Diagnostics for Markov Chain Monte Carlo estimates of the posterior distribution in the Imatinib EC50 and IL12 effect (α) estimated from flow cytometry viability assay using B16F0 cells. The traces for the initial 10000 steps (A and B) and full length (C and D) of three independent chains (colored in black, blue, and red, respectively) with over disperse starting points. (E) The Gelman-Rubin potential scale reduction factor (PSRF) was used to assess convergence of the Markov Chains to the posterior distribution in the Imatinib EC50 (left panel) and IL12 effect α (right panel), where a value of less than 1.2 indicated that the chains have converged to sampling the posterior distribution. The MCMC chains converged after less than 50,000 steps. (F) New steps in the Markov Chain were proposed using a normally distributed random number generator with a mean of zero and adjusted standard deviation such that the acceptance fraction was 0.2. [file 12964_2020_547_MOESM2_ESM.pdf]

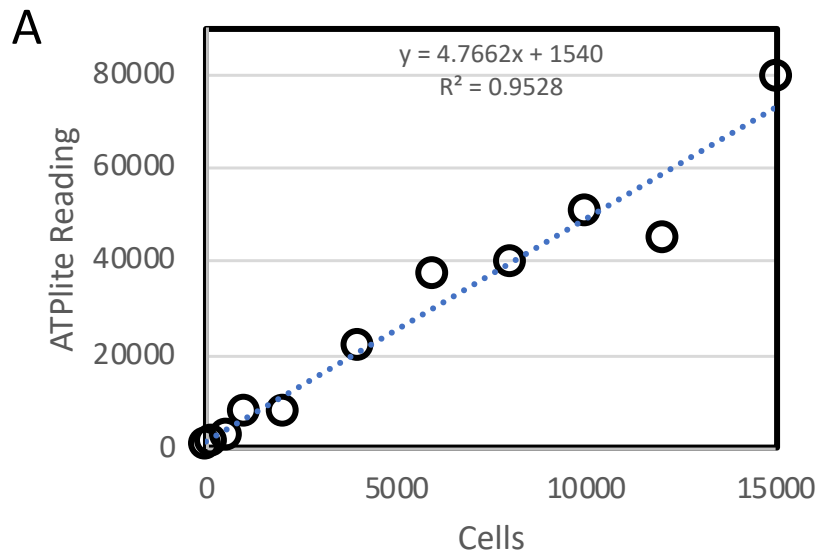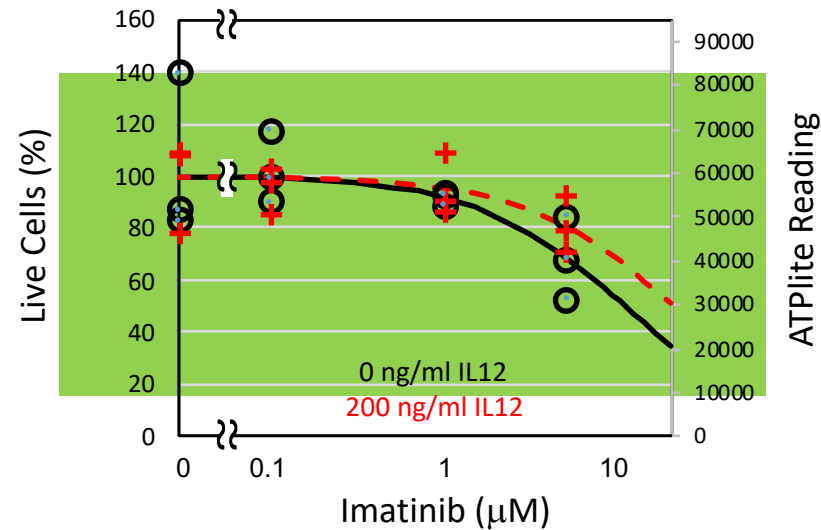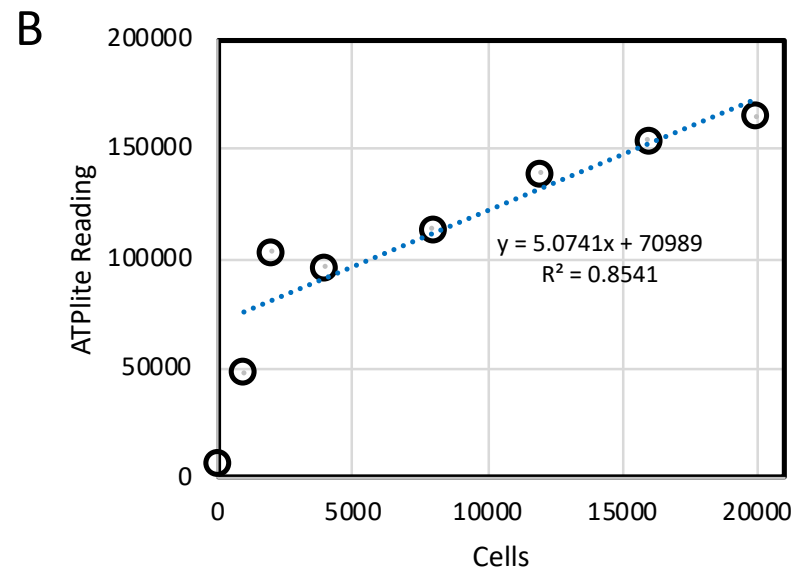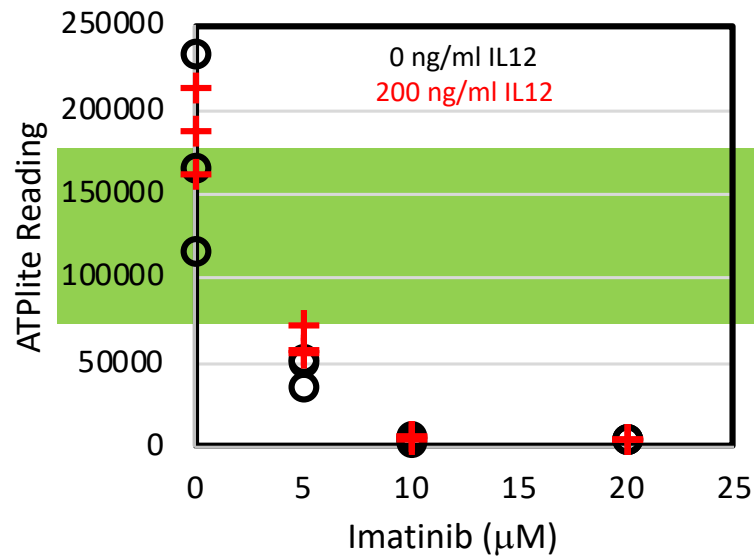

Supplement: Supplementary file 4 — Additional file 3 Supplemental Figure S3: Calibration curves for quantifying cell viability using the ATPlite assay. (A) Increasing concentrations of B16F0 cells were plated just prior to reading viability using the ATPlite assay to establish the dynamic range of the assay (left panel). Results for experimental conditions that were acquired within the dynamic range of the assay are indicated by green overlay. (B) In a separate dose-finding experiment, increasing concentrations of B16F0 cells were plated just prior to reading viability using the ATPlite assay (left panel). While higher doses of imatinib appeared to reduce cell viability to near zero, the experimental conditions were acquired outside of the dynamic range of the assay (green overlay). [file 12964_2020_547_MOESM3_ESM.pdf]

A

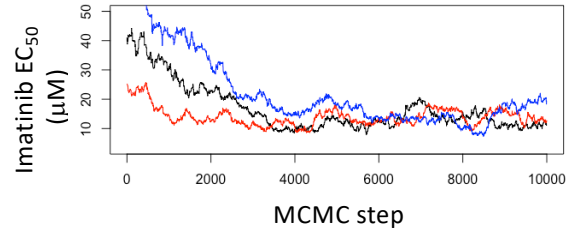

C

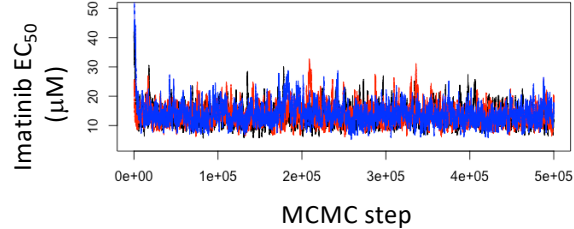

E

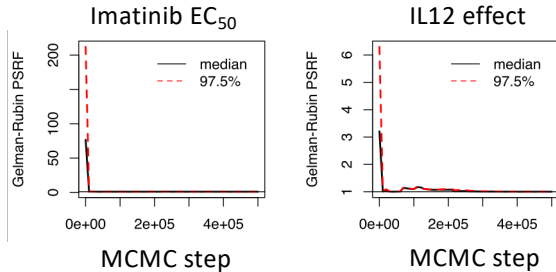

B

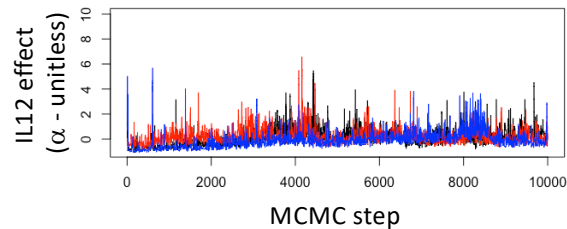

D

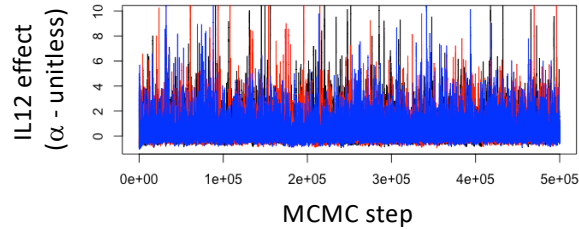

F

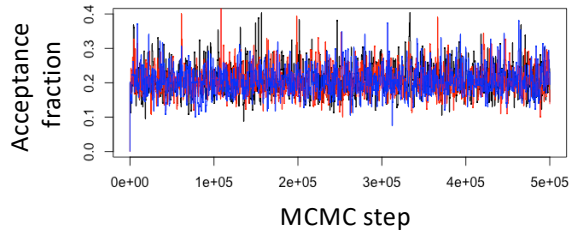

Supplement: Supplementary file 5 — Additional file 4 Supplemental Figure S4: Diagnostics for Markov Chain Monte Carlo estimates of the posterior distribution in the Imatinib EC50 and IL12 effect (α) estimated from ATPlite assay results using Melan-A cells. The traces for the initial 10000 steps (A and B) and full length (C and D) of three independent chains (colored in black, blue, and red, respectively) with over disperse starting points. (E) The Gelman-Rubin potential scale reduction factor (PSRF) was used to assess convergence of the Markov Chains to the posterior distribution in the Imatinib EC50 (left panel) and IL12 effect α (right panel), where a value of less than 1.2 indicated that the chains have converged to sampling the posterior distribution. The MCMC chains converged after less than 50,000 steps. (F) New steps in the Markov Chain were proposed using a normally distributed random number generator with a mean of zero and adjusted standard deviation such that the acceptance fraction was 0.2. [file 12964_2020_547_MOESM4_ESM.pdf]

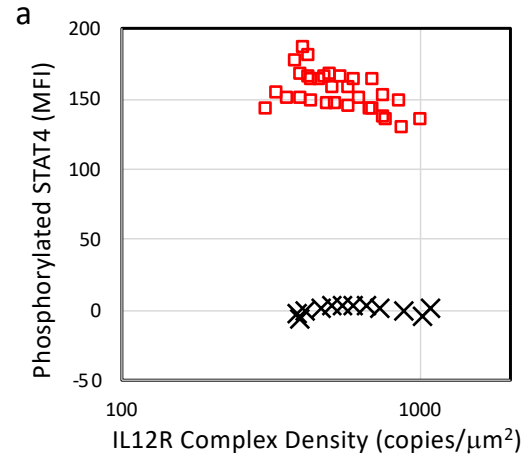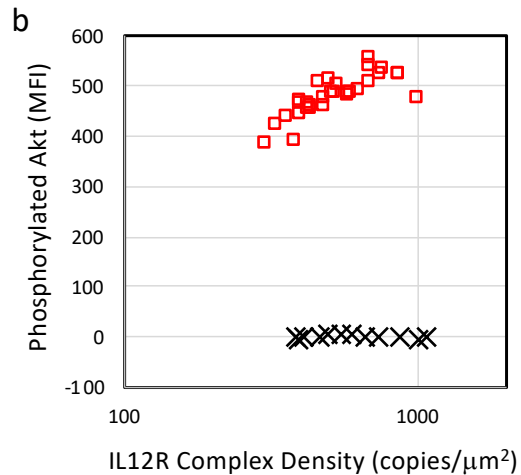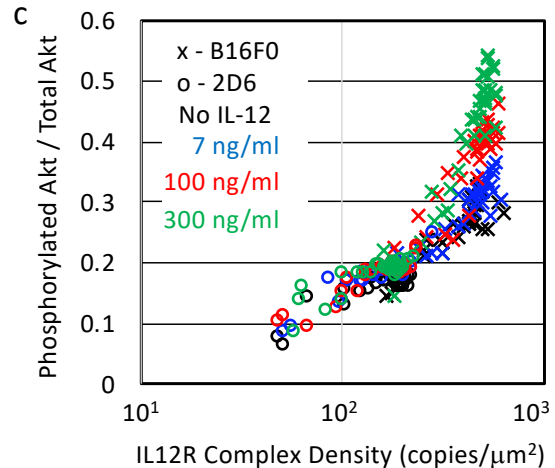

Supplement: Supplementary file 6 — Additional file 5 Supplemental Figure S5: Estimating total Akt and STAT4 values. Media contained serum was used to elicit a near maximal phosphorylation of STAT4 (a) and Akt (b) in B16F0 cells following a 12 hour stimulation (red squares). Phosphorylation of STAT4 and Akt was assayed by flow cytometry, where results are shown for each subgroup based on IL12RB2 expression. Single-stained controls for IL12RB2 in B16F0 (black x’s) cells were used to establish that fluorescence associated with measuring Akt and STAT4 phosphorylation was not due to fluorescent spillover. From these data, we developed a linear relationship between total Akt and IL12RB2 density (Total Akt (MFI) = 0.352 * IL12RB2 (in copies/ μm2) + 382). The total Akt was then estimated for each IL12RB2 subgroup and used for the value of TOTS in equation (12). (c) The ratio of phosphorylated Akt to total Akt, which corresponds to pS/TOTS in equations 12, 14, 16, and 19, was calculated for the different experimental conditions. Total Akt was assumed to follow the same dependence on IL12RB2 in 2D6 and B16F0 cells. [file 12964_2020_547_MOESM5_ESM.pdf]
